# Central nervous system relapse in patients over 80 years with diffuse large B‐cell lymphoma: an analysis of two LYSA studies

**DOI:** 10.1002/cam4.1139

**Published:** 2018-02-23

**Authors:** Aurélie Cabannes‐Hamy, Frederic Peyrade, Fabrice Jardin, Jean‐François Emile, Vincent Delwail, Nicolas Mounier, Corinne Haioun, Aurore Perrot, Olivier Fitoussi, Diane Lara, Richard Delarue, Marc André, Fritz Offner, Hervé Ghesquières, Laurent Pascal, Carole Soussain, Julien Lazarovici, Jean‐Marc Schiano, Philippe Gaulard, Hervé Tilly, Catherine Thieblemont, the LYSA, F Bauduer, C Besson, M Blanc, D Bordessoule, A Bosly, O Casasnovas, S Castaigne, B Coiffier, J Collignon, A Delmer, H Demuynck, A Devidas, O Fain, E Fleck, C Fruchart, J Gabarre, C Gisselbrecht, M Janvier, P Lederlin, G Lepeu, M Macro, M Maerevoet, C Martin, H Orfeuvre, C Rose, B Salles, N Straetmans, E Neste, K Eygen, M Wetterwald, P Zachee, B Anglaret, S Bologna, C Bonnet, D Bron, B Choufi, B Christian, T Connerotte, S Corm, B Corront, H Farhat, C Ferme, S Lefort, F Morschhauser, D Pranger, C Sohn, A Hoof, L Wu, H Zerazhi, Loic Chartier, Laurence Girard, O'Brian Saindoy, Bettina Fabiani, Peggy Dartigues, the lymphoma study association

**Affiliations:** ^1^ APHP Hôpital Saint‐Louis Hemato‐Oncologie Paris France; ^2^ Université Diderot Sorbonne Paris‐Cité Paris France; ^3^ EA7324 Université Descartes Paris France; ^4^ Centre Antoine Lacassagne Hematologie Nice France; ^5^ Department of Hematology Centre Henri Becquerel UNIROUEN INSERMU1245 Rouen France; ^6^ APHP Hôpital universitaire Ambroise Paré Service d'anatomie pathologique Boulogne France; ^7^ Department of Oncology‐Hematology and Cell Therapy University Hospital CIC INSERM 1402 Poitiers France; ^8^ Onco Hématologie CHU L'archet Nice France; ^9^ Lymphoid Malignancies Unit AP‐HP Groupe Hospitalier Mondor Créteil France; ^10^ Hematology Department University Hospital Vandoeuvre Les Nancy France; ^11^ Polyclinique Bordeaux‐Nord Service d'onco‐hématologie Bordeaux France; ^12^ Service d'Hematologie Oncologie Centre Hospitalier de Versailles Le Chesnay France; ^13^ Hematologie APHP Hôpital Necker Paris France; ^14^ Department of Hematology Université catholique de Louvain CHU UCL Namur Yvoir Belgium; ^15^ CHU Department of internal medicine Ghent Belgium; ^16^ Hematologie Centre hospitalier Lyon Sud Université Claude Bernard Lyon 1 Pierre‐Benite France; ^17^ Groupement des Hôpitaux de l'Institut Catholique de Lille Hematologie Lille France; ^18^ Hematologie CLCC Hôpital René Huguenin ‐ Institut Curie Saint‐Cloud France; ^19^ Département d'Hématologie Gustave Roussy Université Paris‐Saclay F‐94805 Villejuif; ^20^ Hematologie Institut Paoli‐Calmettes Marseille France; ^21^ Département de Pathologie Hôpital Henri Mondor AP‐HP Créteil France; ^22^ INSERM U955 Créteil France; ^23^ Université Paris‐Est Créteil France

**Keywords:** Aged 80 and over, CNS relapse, DLBCL, elderly

## Abstract

CNS relapse is reported in 2–5% of diffuse large B‐cell lymphoma (DLBCL) patients, dramatically decreasing overall survival (OS). Very few studies address incidence and risk factors of CNS relapse in very elderly patients, a challenging population to treat given their commonly associated comorbidities. A retrospective analysis was performed of 270 DLBCL patients >80 years treated between 2004 and 2013 in two multicentre phase II LYSA trials (LNH03‐7B, LNH09‐7B) evaluating the addition of rituximab or ofatumumab to mini‐CHOP as front‐line therapy. No patients received CNS prophylaxis. CNS relapse was evaluated according to cumulative incidence, patient characteristics, risk factors, and survival. Median age was 83 years (range: 79–95). After a median follow‐up of 28.7 months, eight patients had CNS relapse (3.0%). Median time between inclusion and CNS relapse was 19.2 months (range: 3.2–32.6). Patients survived a median of 1.5 months after CNS relapse (range: 0.4–4.1). Median OS from relapse was significantly lower in CNS relapse patients (1.5 months, 95% CI: 0.4–3.5) compared to patients with non‐CNS relapse (6.6 months; 95% CI: 4.6–11.9). No baseline characteristics were associated with CNS relapse. The proportion of patients with CNS disease did not differ significantly between patients with low‐intermediate risk according to CNS‐IPI and patients with high risk (3% vs. 2.8%, *P* = 1.00). CNS relapse cumulative incidence in very elderly treatment‐naive patients is 1.8% at 2 years and is associated with poor survival. This population had a long median time to CNS relapse. Absence of prophylaxis did not strongly impact CNS relapse incidence.

## Introduction

Diffuse large B‐cell lymphoma (DLBCL) is the most common lymphoid malignancy worldwide. The prevalence of DLBCL patients aged 80–84 years is reported to be 2.5‐fold higher than that of patients aged 60–64 years 1. Treating very elderly patients is particularly challenging given the likelihood of comorbidities and concerns over risks of toxicity. Poorer survival outcomes are seen in this population despite a clinical presentation and prognostic parameters similar to those of younger patients, with retrospective and prospective studies placing median overall survival (OS) in this older population in the range of 2.0–2.5 years 2, 3, 4. Disease progression is the main cause of death in approximately half of these patients.

Central nervous system (CNS) relapse is one of the most devastating and rapidly fatal complication in DLBCL, which occurs in around 2–5% of DLBCL patients 5, 6, 7, 8, 9, 10, dramatically shortening OS, often to less than 6 months after CNS relapse. Although contentious, several risk factors have been reported to be associated with higher relapse rates, including advanced disease stage (Ann Arbor stage III or IV), elevated lactate dehydrogenase (LDH) levels, Eastern Cooperative Oncology Group (ECOG) performance status (PS) of 2 or more, involvement of more than two extranodal sites, age‐adjusted international prognostic index (aaIPI) of 2 or more, involvement of the testis, breast, or the ovaries (considered organs with immune privilege), or involvement of the base of the skull 11, 12, 13, 14. The CNS international prognostic index (CNS‐IPI) is a validated tool for classifying DLBCL patients into three risk groups for CNS relapse 15. The model uses kidney and/or adrenal gland involvement as well as the IPI. Patients in the low‐ and intermediate‐risk groups are associated with a risk <5% while the high‐risk group is associated with *a* > 10% risk. CNS prophylaxis is generally proposed to high‐risk subgroups. Although there is currently no consensus for the optimal CNS prophylactic strategy, intrathecal or high‐dose intravenous methotrexate is usually used 13, 14, 16, 17.

Most studies reporting incidence and risk factors of CNS relapse concern DLBCL patients under the age of 80 years 18, 19, and little is known about CNS recurrence in the very elderly, aged over 80 years. CNS prophylaxis is rarely implemented in this population due to the burden of comorbidities with frequent antiplatelet or anticoagulant treatment (renal failure, and hypoalbuminemia), as well as the potential toxicity of the prophylaxis. We retrospectively evaluated CNS relapse in very elderly DLBCL patients aged 80 years or older, treated in two prospective LYSA studies with miniCHOP therapy, associated with either rituximab or ofatumumab, another anti‐CD20 monoclonal antibody. CNS relapse incidence, risk factors, and specific survival in this population were analyzed, with the aim of fine‐tuning therapeutic management of this fragile population.

## Patients and Methods

### Clinical studies and patient population

Data were collected retrospectively from two multicentre, phase II, open‐label, single arm LYSA trials conducted in France and Belgium, LNH03‐7B (NCT01087424) between 2004 and 2007 and LNH09‐7B (NCT01195714) between 2009 and 2013. Results of these studies are presented elsewhere 4, 20. Main inclusion criteria for both studies were previously untreated CD20 +  DLBCL (diagnosis of aggressive lymphoma confirmed by an expert hematopathologist panel), follicular lymphoma grade 3B, or *de novo* transformed follicular lymphoma, age over 80 years, Ann Arbor stage of I bulky, II, III or IV, aaIPI ≤ 3, no previous DLBCL treatment, ECOG PS ≤2 for LNH03‐7B or any PS for LNH09‐7B, and recent negative HIV, HBV, and HCV serology. Main exclusion criteria were a history of treated or nontreated indolent lymphoma, CNS or meningeal involvement, creatinine > 150 *μ*mol/L, total bilirubin > 30 mmol/L, transaminases > 2.5 times the upper normal limit (unless disease‐related), neutrophils < 1.5 G/L, or platelets < 100 G/L. All patients signed written informed consent. Studies were approved by an independent research ethics committee and were carried out in accordance with the International Conference on Harmonisation Good Clinical Practice Guidelines, the Declaration of Helsinki, and local regulatory requirements and laws.

### Treatment

Patients in the LNH03‐7B study received rituximab‐miniCHOP (R‐miniCHOP). In the LNH09‐7B study, ofatumumab was associated with miniCHOP (O‐miniCHOP). MiniCHOP consisted of cyclophosphamide (400 mg/m²), doxorubicin (25 mg/m²), and vincristine (1 mg) on day 1 of each cycle and oral prednisone (40 mg/m²) on days 1–5. For LNH03‐7B rituximab (375 mg/m²) was administered on day 1. LNH09‐7B included a pre‐phase of oral prednisone and vincristine, then ofatumumab (1000 mg/m²) on day 1. Six 3‐week cycles were planned. CNS prophylaxis was not recommended and no patients received prophylaxis for CNS relapse. At relapse, diagnosis of CNS disease was based on symptoms and radiological finding including a brain scan and/or MRI.

### Data collection

Data from the initial clinical examination, laboratory tests, chest X‐ray, abdominal sonography, computed tomography of thorax, and abdomen and a bone marrow biopsy were collected, along with follow‐up data for disease progression and survival. Staging was based on thoracic and abdominal computerized scan. Tumor measurements were assessed by the treating physician or local radiologist, and bulky disease was defined as any mass with a maximum diameter of 10 cm or more. Cerebrospinal fluid examination, bone marrow biopsy, and 18Fluorodeoxyglucose PET was not mandatory for staging or for assessment of response.

### Statistical analysis

Cumulative incidence of CNS relapse was calculated using the competing risk method, considering non‐CNS relapse and death without relapse as competing risks. We controlled for the effects of prognostic factors on outcome using the competing risk formulation of Cox model regression which investigates the effect of explanatory variables on different competing events, such as CNS relapse, non‐CNS relapse, or death during course of a disease. Progression‐free survival (PFS) was calculated from the date of diagnosis to the date of progression, relapse, or death from any cause. Overall survival (OS) was calculated from the date of diagnosis to the date of death from any cause. The impact of CNS relapse on PFS and OS analyses was performed in the global population and in a subset of patients with relapse. Survival functions were estimated by the Kaplan–Meier method and compared by the log‐rank test. Comparisons between patients with and without CNS disease were performed by Fisher's exact test for discrete variables and Wilcoxon Mann–Whitney test for continuous variables. Potential predictive covariates of CNS relapse analyzed were sex, Ann Arbor stage, ECOG PS, LDH level, number of extranodal sites, aaIPI, IPI, involvement of specific extranodal sites (breast, gonads, kidney, adrenal, cavum, bone marrow), histological parameters (Hans score, MYC status, and cMYC value), and CNS‐IPI. Analyses were performed with SAS 9.2 (SAS Institute, Cary, NC).

## Results

### Patient characteristics

Our study population concerns the 270 patients included in the two trials, consisting of 150 in the LNH03‐7B treated with R‐miniCHOP and 120 in the LNH0‐97B treated with O‐miniCHOP. Patient characteristics in the overall population are presented in Table 1. Median age was 83 years (range: 79–95), with a female/male sex ratio of 0.65. Patients had generally good status with 69% presenting a PS 0 or 1. LDH levels were elevated in 64% of patients. Most patients (76%) presented with disseminated disease having an Ann Arbor stage III or IV, and 37% had at least two extranodal sites. A total of 17.5% of the 114 evaluated patients had bone marrow involvement, while disease in immune privilege organs (testis, ovaries, and breast) or the base of the skull was rare, occurring in less than 5% of patients.

**Table 1:** Clinical and biological characteristics of the global DLBCL population and according to CNS relapse

	All DLBCL	CNS relapse		*P*‐value^a^
	*N* = 270	No *N* = 262	Yes *N* = 8	
Study				0.74
LNH03‐7B/R‐miniCHOP	150 (55.6%)	145 (55.3%)	5 (62.5%)	
LNH09‐7B/O‐miniCHOP	120 (44.4%)	117 (44.7%)	3 (37.5%)	
Age (years)				0.66
Median (range)	83 (79–95)	83 (79–95)	83.5 (80–87)	
Sex				0.27
Male	106 (39.3%)	101 (38.5%)	5 (62.5%)	
Female	164 (60.7%)	161 (61.5%)	3 (37.5%)	
Performance status (ECOG)				0.71
<2	185 (68.5%)	180 (68.7%)	5 (62.5%)	
≥2	85 (31.5%)	82 (31.3%)	3 (37.5%)	
LDH				1.00
≤Normal	98 (36.3%)	95 (36.3%)	3 (37.5%)	
>Normal	172 (63.7%)	167 (63.7%)	5 (62.5%)	
Ann Arbor stage				1.00
I–II	65 (24.1%)	63 (24.0%)	2 (25.0%)	
III–IV	205 (75.9%)	199 (76.0%)	6 (75.0%)	
IPI				1.00
0–2	82 (30.4%)	80 (30.5%)	2 (25.0%)	
3–5	188 (69.6%)	182 (69.5%)	6 (75.0%)	
Extranodal sites				
Breast	13 (4.8%)	13 (5.0%)	0 (0.0%)	1.00
Gonads	9 (3.3%)	9 (3.4%)	0 (0.0%)	1.00
Kidney	10 (3.7%)	10 (3.8%)	0 (0.0%)	1.00
Adrenal	5 (1.9%)	5 (1.9%)	0 (0.0%)	1.00
Cavum	6 (2.2%)	5 (1.9%)	1 (12.5%)	0.22
Bone marrow (*N* = 114)	20 (17.5%)	20 (18.5%)	0 (0.0%)	0.67
Number of extranodal sites				0.71
<2	171 (63.3%)	165 (63.0%)	6 (75.0%)	
≥2	99 (36.7%)	97 (37.0%)	2 (25.0%)	
Age‐adjusted IPI				0.71
0–1	103 (38.1%)	101 (38.5%)	2 (25.0%)	
2–3	167 (61.9%)	161 (61.5%)	6 (75.0%)	
CNS‐IPI				
Low risk (0–1 factors)	33 (12.3%)	32 (12.3%)	1 (12.5%)	1.00
Intermediate risk (2–3 factors)	165 (61.3%)	160 (61.3%)	5 (62.5%)	
High risk (4–6 factors)	71 (26.4%)	69 (26.4%)	2 (25.0%)	

LDH: Lactate dehydrogenase, aaIPI: age‐adjusted International Prognostic Index.

a CNS versus no‐CNS relapse populations were compared with Fisher's exact or Wilcoxon Mann–Whitney (for age) tests. Missing data for 1 to 10 patients for each parameter unless otherwise indicated.

### Cumulative incidence, risk factors, and outcome of patients with CNS relapse

**Figure 1:**
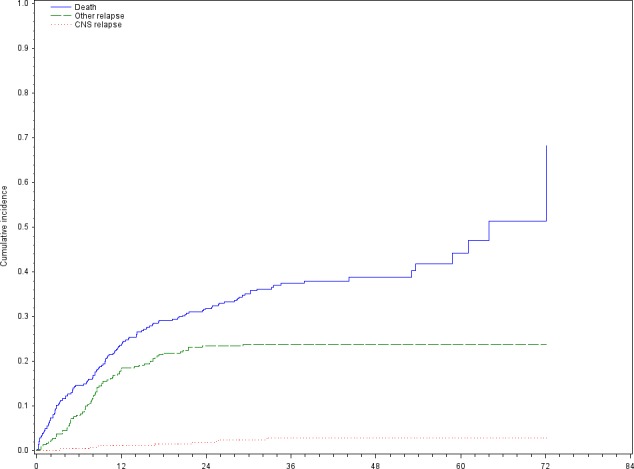
Cumulative incidence of CNS relapse (N = 8), non‐CNS relapse (*N* = 71), and death (*N* = 125) adjusted on IPI score in the global DLBCL population.

On‐study data for CNS relapse and follow‐up were not collected for one patient. After a median follow‐up of 28.7 months (range: 0.1–72.1), eight cases of CNS relapse (3.0%) were reported among the 269 patients analyzed (3.0%), five of whom who were receiving R‐miniCHOP and three were receiving O‐miniCHOP. Median time between inclusion and CNS relapse was 19.2 months (range: 3.2–32.6). The estimated 1‐year and 2‐year cumulative incidence of CNS relapse was 1.16% and 1.80%, respectively (Fig. 1). As other relapse is considered as a competing event for CNS relapse, all the eight CNS relapse cases reported here are in first relapse.

**Figure 2:**
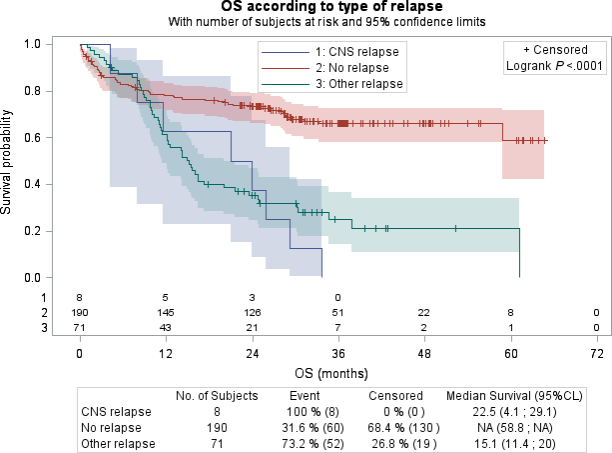
Kaplan–Meier analysis of overall survival in patients without relapse and in patients with CNS relapse and with non‐CNS relapse, with number of subjects at risk and 95% confidence limits.

Patients with CNS relapse had significantly worse survival than those without relapse, with a median OS of 22.5 months (95% CI: 4.1–29.1) compared to median not reached (95% CI: 58.8 months to not reached), respectively, and a hazard ratio of 3.77 (95% CI: 1.80–7.90, *P* = 0.004; Fig. 2).Two‐year OS was 37.5% for patients with CNS relapse compared to 73.3% for patients without relapse (Fig.**** 2).

CNS‐IPI classified 33 (12%) patients in the low‐risk group, 165 (61%) in the intermediate‐risk group, and 71 (26%) in the high‐risk group. The proportion of patients with CNS disease did not differ significantly between patients with low‐intermediate risk and patients with high risk according to CNS‐IPI (3% vs. 2.8%, *P* = 1.00). No significant prognostic factor was identified for CNS relapse according to competing risk method.

### Clinical profile of CNS relapse patients at lymphoma diagnosis

Taking into consideration, the relatively small number of patients with CNS relapse, clinical presentation at diagnosis of this subpopulation did not differ significantly from patients without CNS relapse for any of the parameters analyzed (Table 1). Median age was 83.5 years in the CNS relapse group. Five were male, six had disseminated disease, and high LDH levels were reported in five patients. Two patients had two or more extranodal sites, none of which involved immune privilege organs. One patient had cavum involvement. Six patients had an aaIPI of 2 or 3, while CNS‐IPI was 1 (low risk) for one patient, 2–3 (intermediate risk) for five patients, and 4 (high risk) for two patients. Six presented non‐germinal centre B‐cell‐like (non‐GCB) DLBCL, one had GCB DLBCL and data were missing for one patient. By comparison, among the 133 evaluable samples in the global population, 54.9% (73/133) were non‐GCB DLBCL and 33.8% (45/133) were GCB. MYC expression was positive in between 30% and 50% of the cells in all four available samples, whereas among the 58 samples available for the global population, 53.5% (31/58) had ≥ 40% expression.

### Description of the CNS relapse

Neurological symptoms at CNS relapse were either mild including loss of autonomy, asthenia, hearing impairment, urinary incontinence, or more prominent with delirium, aphasia, intracranial hypertension, or consciousness disorder (Table 2). Of the eight patients, all except one (with one missing data) presented at CNS relapse with ECOG PS ≥ 2. One patient had elevated LDH levels (missing data for two patients), all patients had exploratory imaging with a brain CT‐scan, and three had a brain MRI, one of whom also had a lumbar puncture. All CNS relapses were parenchymal with no reports of meningeal disease, despite the very few number of completed lumbar puncture. Treatment of CNS relapse was proposed to five patients, entailed supportive care with corticosteroids in two patients, radiation therapy alone in one patient, radio‐chemotherapy with rituximab/temodal (five cycles) in one patient, and chemotherapy alone including rituximab/aracytine/vepeside (two cycles) in another. All eight patients progressed and died within 3 months.

**Table 2:** Clinical and biological characteristics of patients with CNS relapse, at inclusion and at CNS relapse diagnosis

Patient	1	2	3	4	5	6	7	8
Characteristics at inclusion								
Study	LNH03‐07B	LNH03‐07B	LNH03‐07B	LNH03‐07B	LNH03‐07B	LNH09‐07B	LNH09‐07B	LNH09‐07B
Sex	Male	Male	Male	Female	Female	Male	Female	Male
Age at diagnosis (years)	88	81	86	86	81	83	80	88
LDH	≤N	>N	≤N	≤N	>N	>N	>N	>N
ECOG PS	≥2	≥2	<2	<2	<2	<2	≥2	<2
N extranodal sites	<2	<2	<2	<2	<2	≥2	<2	≥2
Involved sites	None	Cavum	Left cheek	None	None	Spleen, Lung, Colon	Lung	Spleen, Pericardium
Ann Arbor Stage	III–IV	I–II	I–II	III–IV	III–IV	III–IV	III–IV	III–IV
IPI	3–5	3–5	0–2	0–2	3–5	3–5	3–5	3–5
aaIPI	2–3	2–3	0–1	0–1	2–3	2–3	2–3	2–3
Kidney	Normal	Normal	Normal	Normal	Normal	Normal	Normal	Normal
Adrenal	Normal	Normal	Normal	Normal	Normal	Normal	Normal	Normal
BCL2 (%)	90	Missing	90	100	Missing	Missing	Missing	Missing
MYC (%)	30	40	50	40	Missing	Missing	Missing	Missing
CNS‐IPI	2	2	1	2	3	4	3	4
Hans score	Non‐GC	Non‐GC	Non‐GC	Non‐GC	Missing	GC	Non‐GC	Non‐GC
Response to first treatment	CR	CR	PD	CR	PD	PR	CR	CR
Characteristics at CNS relapse								
Age at relapse (years)	89	83	88	86	81	85	82	89
Time to CNS relapse (years)	0.6	2.1	2.1	0.7	0.3	2.7	1.8	1.4
Relapse pattern	Parenchymal	Parenchymal	Parenchymal	Parenchymal	Parenchymal	Parenchymal	Parenchymal	Parenchymal
CNS relapse only	Yes	Yes	No	No	Yes	No	Yes	No
Clinical symptoms	Delirium	Consciousness disorder, aphasia	Aphasia, loss of autonomy, incontinence	Dizziness, intracranial hypertension, asthenia	Disorientation	Cognitive then consciousness disorder	Right hemiparesis	Blurred vision, hearing impairment
CT‐scan	Yes	Yes	Yes	Yes	Yes	Yes	Yes	Yes
MRI	No	No	Yes	No	No	No	Yes	Yes
Lumbar punction	No	No	No	No	No	No	No	Yes
ECOG PS	≥2	≥2	≥2	≥2	≥2	Missing	≥2	<2
LDH	Missing	≤ N	≤ N	Missing	≤N	>N	≤N	≤N
Ann Arbor Stage	III–IV	III–IV	III–IV	III–IV	III–IV	III–IV	I–II	III–IV
aaIPI	2–3	2–3	2–3	2–3	2–3	2–3	0–1	0–1
CNS relapse treatment	None	Radiotherapy (1 session)	Rituximab‐ Temodal (5 cycles)	Rituximab‐ Aracytine‐ VP16‐Dexamethasone (2 cycles)	None	Corticosteroids	None	Corticosteroids
Status after CNS relapse treatment	PD	PD	PD	PD	PD	PD	PD	PD
Survival after CNS relapse (days)	11	13	106	86	28	30	64	133

CNS, central nervous system; aaIPI, age‐adjusted International Prognosis Index; CR, complete response; LDH, lactate dehydrogenase; MRI, magnetic resonance imaging; N, normal; PD, progressive disease; PS, performance status.

### Outcome comparison between patients with CNS relapse and patients with non‐CNS relapse

Among the 270 patients, 71 (26.3%) patients presented non‐CNS relapse. Median time between inclusion and non‐CNS relapses was 8.1 months (range: 0.4–29.2 months). Cumulative incidence of non‐CNS relapse was 18.1% at 1 year and 23.4% at 2 years.

**Figure 3:**
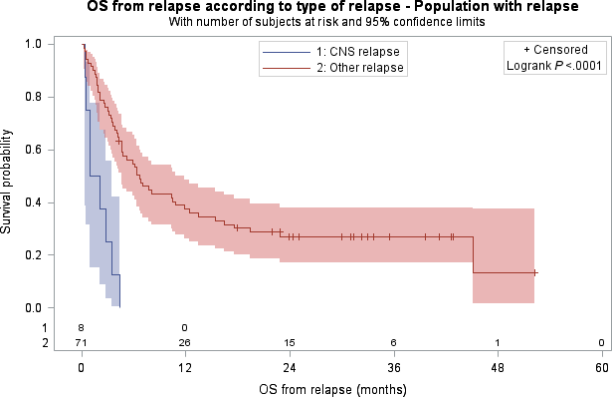
Kaplan–Meier analysis of specific overall survival after relapse for patients with CNS relapse and for patients with non‐CNS relapse, with number of subjects at risk and 95% confidence limits.

When comparing CNS and non‐CNS patients, no difference was observed between baseline characteristics of patients with CNS relapse and patients with non‐CNS relapse. Occurrence of CNS relapses and non‐CNS relapses were observed in 50% and 74.6% of the patients within the first year after inclusion, and in 75% and 97.2% of patients within the two first years after inclusion, respectively. Neither OS nor PFS was significantly different between the patients with CNS relapse compared to the patients with non‐CNS relapse, with a median OS of 22.5 months versus 15.1 months, respectively, (HR = 0.79, *P* = 0.53), and a median PFS of 12.1 months versus 8.1 months, respectively, (HR = 0.49, *P* = 0.07). However, patients with CNS relapse had significantly worse survival from time of relapse than those with non‐CNS relapse, with a median OS from relapse of 1.5 months (95% CI: 0.4–3.5) compared to 6.6 months (95% CI: 4.6 –11.9), respectively, and a hazard ratio of 5.29 (95% CI: 2.36–11.88, *P* < 0.001; Fig. 3).

## Discussion

Patients younger than 80 years are more likely to respond to and tolerate standard treatments than their older counterparts 18, 19. However with the number of older DLBCL patients steadily increasing, identifying the treatment strategy optimally adapted to this typically fragile population and minimizing unnecessary treatment administration, is an important issue to address, particularly for specific cases such as CNS relapse. To the best of our knowledge, this is the first evaluation of CNS relapse in very elderly patients diagnosed with aggressive non‐Hodgkin lymphoma. In this retrospective analysis of a cohort of 270 patients treated with miniCHOP combined with either rituximab or ofatumumab having a median age of 83 years (range: 79–95), CNS relapse was reported in 3% of the population after a median follow‐up of nearly 2.5 years. This incidence in an elderly population falls within the range of published values, most of which include younger populations 5, 7, 8, 9, 10, 21, 22. CNS disease in elderly patients is particularly sticky to diagnose, given the frequently poor neurological symptoms and many confounding factors at this age of life, but the fine‐tuned data collection in these prospective studies may have optimized CNS symptoms detection, and this value should reflect the real incidence of CNS relapse in elderly population. It is of note that none of the 270 patients received CNS prophylaxis, which is driven by the absence of specific recommendations and the ubiquitous presence of comorbidities in this age group. Nonetheless, in this small population, the absence of prophylaxis did not appear to impact incidence of CNS relapse.

Among the eight patients with CNS relapse, five had elevated LDH levels, two had at least two extranodal sites, and six had aaIPI of 2 or 3—all of which have been identified as risk factors in younger populations 5, 7, 8, 9, 10, 21, 22. Our analysis showed that incidence of these factors was not significantly different in patients with CNS relapse compared to those without. Savage et al. reported an increased likelihood of CNS relapse in MYC + DLBCL 23. Although all four CNS relapse patients with MYC data showed MYC positivity, limited conclusions can be drawn with this small number of patients. Finally, CNS‐IPI did not appear to be a predictor of CNS relapse in this elderly population, with equivalent incidences in patients categorized as low risk or intermediate risk compared to those with high risk (3.0% vs. 2.8%), in contrast to the 0.8% for the low‐risk, 3.9% for the intermediate‐risk, and 12.0% for the high‐risk groups reported in the validation cohort in the study by Schmitz et al. 15. On one hand this leaves the relevance of this scale in older populations open to debate; given that the median age in the training cohort of their study was 58 years (18–80), while the validation cohort had a median age of 65 years (range: 16–94). On the other hand, it highlights the limitation of the conclusions that can be drawn given the relatively small number of patients in our study. The neurologic symptoms reported in these older patients did not differ noticeably from those reported in younger patients.

Published results from the LNH03‐7B study support a survival advantage by associating low‐dose chemotherapy (miniCHOP) with standard rituximab in selected very elderly DLBCL patients 4. Preliminary results with ofatumumab suggest a similar scenario for this alternative immunotherapy when associated with miniCHOP 20. Nonetheless, these treatment regimens did not appear to have a dramatic impact on the effect of CNS relapse, with median OS dropping dramatically from median not reached (95% CI: 58.8 months to not reached) in patients with no relapse, with a 3.8‐fold increased likelihood of death with CNS relapse and a median OS of only 22.5 months (95% CI: 4.1–29.1). Given the small patient number in this study, it is difficult to interpret the proportion of CNS relapse according to treatment type, with 3.3% in the R‐miniCHOP group versus 2.5% with O‐miniCHOP. Similarly in the literature, this is open to debate with Feugier et al. reporting that the addition of rituximab to CHOP did not influence the risk of CNS occurrence 5, while Boehme et al. reported the inverse situation with CHOP‐14^7^.

In our study, the median time between inclusion and CNS relapse of 19.2 months (range: 3.2–32.6) was remarkably long compared to several other studies, which report median durations ranging from 4.7 to 8.0 months 5, 7, 8, 9, 10, 21, 22. One possible explanation is that this reflects the impact of the treatment combination R‐miniCHOP improving OS in the overall very elderly population 4. Nonetheless, this benefit does not appear to impact survival after CNS diagnosis, with death occurring after a median of 1.5 months (range: 0.4–4.1) which is shorter than for other younger populations, ranging from 2.4 to 5.0 months 5, 7, 8, 9, 10, 21, 22. One interesting outcome of our study was the absence of difference in the OS when comparing the “CNS relapse” group and the “non‐CNS relapse” group. Our analysis showed that any relapse, irrespective of its site, has a dramatic effect on OS.

An obvious limitation of this study is the relatively small sample size and the correspondingly small number of patients experiencing CNS relapse, reflecting the difficulty of recruiting patients of this age. It is also likely that the patient population recruited for a clinical trial does not accurately reflect the patient population seen in routine consultations, thereby introducing a bias which in turn offers better outcomes. In keeping with this, it is important to note that our population was in relatively good condition for this age group, with two‐thirds of the population having a PS 0‐1 as well as no or only a single extranodal site of disease. These factors may also contribute to the above‐mentioned long time between inclusion and CNS relapse and similar outcomes irrespective of site of relapse.

In conclusion, with the absence of prophylaxis not having a dramatic impact on incidence, prophylaxis can be avoided in the very elderly given the potential for a negative impact of the associated toxicities. Accumulating data from more elderly patients is warranted to identify risk factors for CNS relapse in this population. In the future, new treatment approaches in the elderly including lenalidomide, currently tested in this population of patients by the LYSA (NCT02128061) or ibrutinib, may be efficient in elderly lymphoma patients experiencing CNS relapses.

## Conflict of Interest

Authors have nothing to disclose related to the purpose of this manuscript.
